# Mapping Inequities in Digital Health Technology Within the World Health Organization’s European Region Using PROGRESS PLUS: Scoping Review

**DOI:** 10.2196/44181

**Published:** 2023-04-28

**Authors:** Katherine E Woolley, Diana Bright, Toby Ayres, Fiona Morgan, Kirsty Little, Alisha R Davies

**Affiliations:** 1 Research and Evaluation Division Public Health Wales Cardiff United Kingdom; 2 National Centre for Population Health and Well-being Research Wales United Kingdom; 3 Evidence Service, Public Health Wales Cardiff United Kingdom

**Keywords:** digital health, health inequities, PROGRESS PLUS, health services accessibility, health care disparities, mobile phone

## Abstract

**Background:**

The use of digital technologies within health care rapidly increased as services transferred to web-based platforms during the COVID-19 pandemic. Inequalities in digital health across the domains of equity are not routinely examined; yet, the long-term integration of digitally delivered services needs to consider such inequalities to ensure equitable benefits.

**Objective:**

This scoping review aimed to map inequities in access, use, and engagement with digital health technologies across equity domains.

**Methods:**

We searched 4 electronic databases (MEDLINE, ASSIA, PsycINFO, and Scopus) for quantitative and mixed methods reviews and meta-analyses published between January 2016 and May 2022. Reviews were limited to those that included studies from the World Health Organization’s European region. Extracted data were mapped against Cochrane’s PROGRESS PLUS (place of residence, race, ethnicity, culture, and language, occupation, gender and sex, religion, education, socioeconomic status, social capital, and other characteristics) dimensions of equity.

**Results:**

In total, 404 unique citations were identified from the searches, and 2 citations were identified from other sources. After eligibility assessment, 22 reviews were included. Consistent evidence was found showing higher *access* to digital health technologies among patients who were of White ethnicity, were English speaking, and had no disability. There were no reviews that explored differences in *access* to digital health care by age, gender and sex, occupation, education, or homeless or substance misuse. Higher *use* of digital health technologies was observed among populations that were White, English speaking, younger, with a higher level of education, of higher economic status, and residents in urban areas. No clear evidence of differences in the *use* of digital technologies by occupation, gender and sex, disability, or homeless or substance misuse was found, nor was clear evidence found in the included reviews on inequalities in the *engagement* with digital technologies. Finally, no reviews were identified that explored differences by place of residence.

**Conclusions:**

Despite awareness of the potential impact of inequalities in digital health, there are important evidence gaps across multiple equity domains. The development of a common framework for evaluating digital health equity in new health initiatives and consistency in reporting findings is needed.

## Introduction

### Background

The use of digital health care has been increasing over the past decade [1], with a rapid acceleration in 2020 as services transferred to web-based platforms during the COVID-19 pandemic [2]. When digital health care is provided with appropriate infrastructure, training, and engagement, it has the potential to improve population health by using artificial intelligence, big data, and precision medicine [3]. Digital health care is especially useful where there is increasing demand and limited resources [4]. However, the use of digital technologies within health care provision (eg, telehealth, eHealth, and artificial intelligence), termed digital health, can contribute to health inequity due to systematic disparities and social determinants of health needs and the ability to engage with digital platforms [5].

The development of digital technologies and digital skills initiatives for patients is beginning to appear in local, national, and global policy and practice strategies, one of which is the World Health Organization (WHO) global strategy to strengthen the international approach to the implementation of digital health [6]. However, an understanding of the underlying barriers to digital technologies and embedding mitigation strategies at an early stage is needed if policy implementation is to be effective. For example, despite digital skills being on the European Commission’s agenda since 2016 [7], in 2021, the majority (54%) of the European Union’s population (aged 16-74 years) had limited basic digital skills, and 14% had none at all [8], with variations present by age group, education level, employment status, and rurality. The extent of the digital inequalities and the need for digital skills in Europe became even more apparent during the COVID-19 pandemic due to the reliance on digital technologies for social support and economic functions, including access to health services and public health systems [2,4].

### Key Components of Digital Health

Digital health equity can be explored through an individual’s ability to access, use, and engage with digital health technology [9]:

- *Access* is defined as the ability to access the technology and other resources required for digital health (eg, digital devices, internet connection, web-based tools, and financial resources).
- *Use* reflects having the skills, digital literacy, and ability to navigate and use digital health technologies.
- *Engagement* refers to the variations that will occur in the level of engagement with digital health technology by individuals. For instance, some individuals may have access and the skills to use digital technology but may choose not to engage with a digital health service.

There is some evidence of digital inequities within Europe [2,5,9]. This is generally reported for older individuals, rural communities, and women and is attributed to a lack of knowledge, opportunities, skill, inaccessibility [10], fear of discrimination, and concern for the cessation of face-to-face service [11,12]. However, there is limited evidence of factors contributing to digital health exclusion for subgroups of the population within the 3 components of digital health (access, use, and engagement) and in assessing the interconnected nature of social categorizations, termed intersectionality. Failure to acknowledge and account for all dimensions of equity within digital health, including the role of intersectionality, can lead to widening health inequities.

Understanding the role of inequity in people’s ability to access, use, and engage with digital health is necessary to ensure that digital health does not cause greater health inequities. Therefore, the aim of this scoping review was to map inequities in digital health technology across different equity domains to help inform future developments toward integrating digital technology into health care practices, systems, and policy within the WHO European region.

## Methods

### Overview

This scoping review used the methodology outlined by Arksey and O’Malley [13]. It comprises the following stages: (1) identifying the research question; (2) identifying relevant studies; (3) study selection; (4) data extraction and analysis; and (5) collating, summarizing, and reporting the results. The PRISMA-ScR (Preferred Reporting Items for Systematic Reviews and Meta-Analyses extension for Scoping Reviews) checklist was used to guide the reporting of the scoping review [14].

We conceptualized inequalities using the PROGRESS PLUS framework [15]. The framework, first developed by Evans and Brown and further adapted by the Campbell and Cochrane Equity Methods Group [16], indicates the different characteristics in which health inequities may be experienced. The acronym stands for place of residence, race, ethnicity, culture, language, occupation, gender and sex, religion, education, socioeconomic status, social capital, and other characteristics *PLUS* (eg, disability, age, and sexual orientation). On the basis of the collected data from the included reviews, the *PLUS* category was subcategorized further into age, disability or complex health needs, and homeless or substance misuse. In addition, for the purpose of this review, religion was combined with race, ethnicity, culture, and language.

### Search Strategy

The MEDLINE, ASSIA, PsycINFO, and Scopus databases were searched for reviews and meta-analyses published between January 2016 and May 2022 to capture the most recent evidence of evolving technologies and changes in health provision resulting from the COVID-19 pandemic. Included search terms related to “Digital Health” AND “Inequities” AND “Review” (Multimedia Appendix 1). A further hand search of key relevant journals (eg, *JMIR* and *Lancet Digital Health*), gray literature databases (eg, the Health Management Information Consortium and the Turning Research into Practice databases), and citation tracking of included literature was performed to identify additional records. Once the data had been extracted and thematically mapped, a further search was undertaken for recent primary research (2018-2022) conducted in WHO Europe countries for equity domains where evidence from reviews could not be extracted. For this step, the search terms to cover “reviews” that were included in the initial search were omitted. Additional inclusion and exclusion criteria remained the same.

### Inclusion and Exclusion Criteria

Studies were eligible for inclusion if they (1) contained a digital technology that connected an individual to a health care professional or service; (2) documented equity through a lens of access, use, and engagement; (3) contained research from countries within the WHO’s European region or was global in interpretation [17]; (4) reported quantitative results; and (5) had a systematic, scoping, rapid, or mapping review methodology (Table 1).

**Table 1:** Inclusion and exclusion criteria.

Characteristics	Included	Excluded
Population	Any	N/A^a^
Concept	Digital health specific The technology connects an individual to health professionals Addressing equity through access to, use of, or engagement with digital health technologies within groups of interest	Technology that connects “peers-to-peers” or “health professional to health professional” (eg, a laboratory providing blood test result to a doctor) If the topic is general “wellness” rather than health (eg, wellness apps)
Context	WHO^b^ Europe countries specific or global in interpretation	Reviews specific to a non–WHO Europe country (eg, US-specific reviews)
Type of evidence	Quantitative or mixed methods systematic, scoping, rapid, or mapping reviews and meta-analyses reporting clear quantitative results were included.	Qualitative reviews Mixed method reviews where quantitative outcomes could not be disaggregated
Language	All languages	N/A

^a^N/A: not applicable.

^b^WHO: World Health Organization.

### Study Selection

Screening by title and abstract and subsequent full paper review of relevant literature was carried out by 3 reviewers (TA, DB, and KEW). Before screening and full paper review, we followed a calibration process against the inclusion and exclusion criteria and the feasibility of disaggregating quantitative results into 10 equity domains and 3 components of digital health (access, use, and engagement) that we were interested in reporting. A second, and in some cases, a third reviewer resolved any uncertainty throughout the screening and full paper review process.

### Data Extraction and Analysis

Two reviewers (TA, DB, or KEW) conducted data extraction independently. Any dispute was solved by consulting a third reviewer (TA or KEW). The form captured information including (1) author and publication date; (2) participant characteristics; (3) interventions and exposures; (4) included study features; (5) equity outcomes; and (6) solutions, limitations, and evidence gaps.

Scoping reviews do not critically appraise the literature, but where this information was available in the included reviews, it was captured. The results were then thematically mapped using the PROGRESS PLUS framework [15] to examine the role of equity within the key components of digital health: access, use, and engagement, in addition to looking for any evidence of intersectionality across the PROGRESS PLUS equity domains. This ensured that socially stratifying factors were considered throughout the review process [15]. The thematically mapped data were summarized using a heat map for each equity domain, with a potential direction of effect being interpreted, where at least 75% of the included reviews found consistent evidence of equity or inequity.

## Results

### Included Reviews

**Figure 1:**
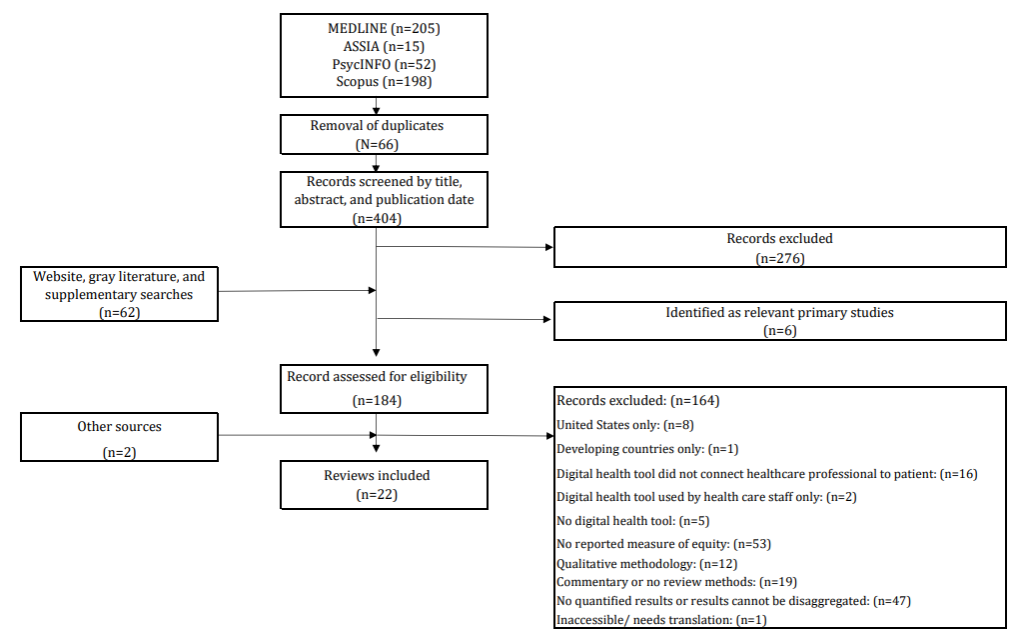
PRISMA (Preferred Reporting Items for Systematic Reviews and Meta-Analyses) flow diagram.

The database searches yielded 404 unique results, with a further 62 obtained from hand searches of journal websites and gray literature, and 2 from other sources, leading to the inclusion of 22 reviews (Figure 1).

### Review Characteristics

**Figure 2:**
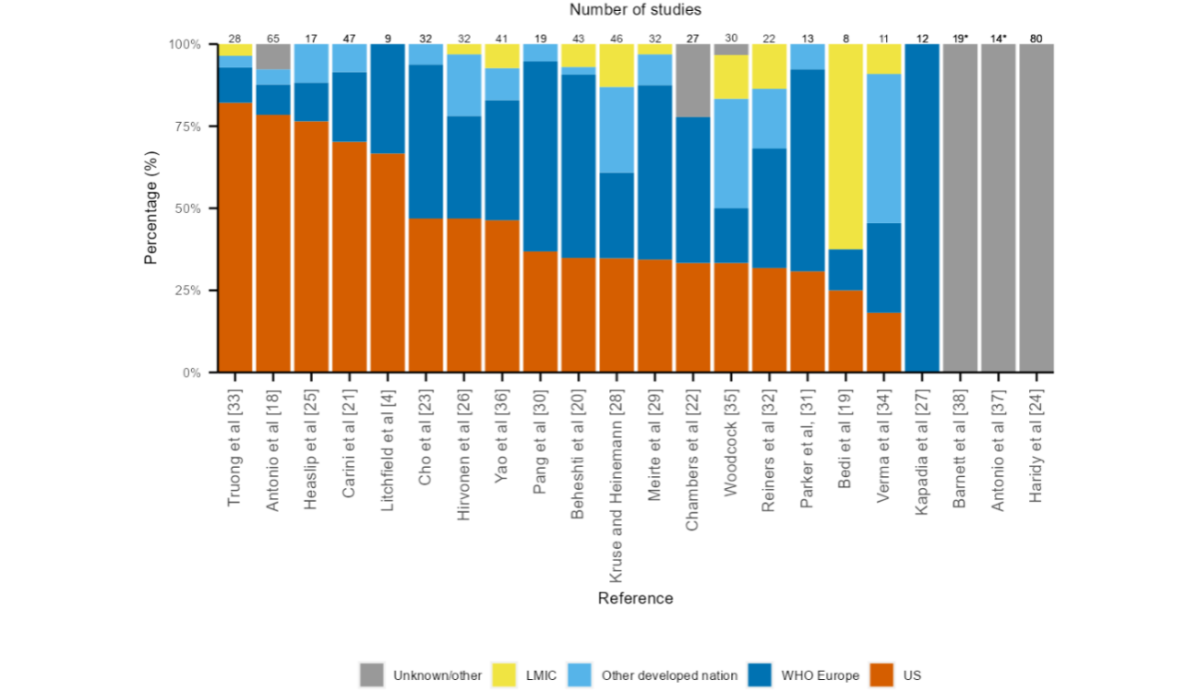
Geographic distribution and number of primary evidence from each of the included reviews. LMIC: low- and middle-income country; US: United States; WHO: World Health Organization. *Geographic information could not be obtained for 3 reviews as they were reviews of reviews (n=2) or not reported (n=1; Table 2).

The primary studies included in this scoping review were mainly from the United States (258/520, 49.4%), followed by Europe (177/520, 33.9%), other developed countries (eg, Australia and Canada; 59/520, 11.3%), and a minority from low- and middle-income countries (LMICs; 20/520, 5.4%; Figure 2). The characteristics of the included reviews are presented in Table 2.

**Table 2:** Characteristic of included reviews.

Review			Inclusion criteria for review									Features of included studies^a^				
			Population		Digital tool		Setting or context		Date limit		Number of studies			Countries		Study design
**Reviews of primary studies**																
	Antonio et al [18]	General population		Tethered patient portals		Not stated		No date-2018		65			United States (n=51) WHO^b^ Europe (n=6) Netherlands (n=4) United Kingdom (n=2) Other developed (n=3) New Zealand (n=1) Australia (n=2) N/A^c^ (n=5)		Quantitative (n=24) Qualitative (n=17) Mixed methods (n=15) Gray literature (n=9)	
	Bedi et al [19]	Children undergoing clef-palette treatment		Telehealth		Not stated		1995-2020		8			United States (n=2) United States and Mexico (n=2) WHO Europe (n=1) Scotland (n=1) LMIC^d^ (n=5) India (n=3) Ecuador (n=1) Brazil (n=1)		Quantitative (n=8) Case series (n=3) Case control (n=3) Case report (n=1) Cohort study (n=1)	
	Beheshti et al [20]	Not stated		Telehealth		Primary care		2000-2018		43			United States (n=15) WHO Europe (n=24) United Kingdom (n=12) Spain (n=1) Germany (n=1) Netherlands (n=3) Greece (n=1) Ireland (n=1) Belgium (n=1) Poland (n=2) Italy (n=1) Sweden (n=1) Other developed (n=1) China (n=1) LMIC (n=3) Bahrain (n=1) Brazil (n=1) Zambia (n=1)		Quantitative (n=40) RCT^e^ (n=14) Observational (n=17) Cross-sectional (n=3) Longitudinal survey (n=1) Descriptive (n=2) Retrospective (n=1) Prospective (n=1) Controlled trial (n=1) Qualitative (n=1) Mixed methods (n=1) Not mentioned (n=1)	
	Carini et al [21]	Not stated		Not stated		Digital patient portals		2013-2019		47			United States (n=33) WHO Europe (n=10) Netherlands (n=3) Finland (n=2) United Kingdom (n=2) France (n=1) Israel (n=1) Sweden (n=1) Other developed (n=4) Canada (n=3) Australia (n=1)		Quantitative (n=39)^f^ Descriptive (n=17) Observational (n=14) Interventional (n=5) RCT (n=3) Qualitative (n=7) Mixed methods (n=2)	
	Chambers et al [22]	General population		Web-based digital service for addressing symptoms, health advice and direction to appropriate services. Excluded treatment services (eg, Cognitive Behavioral Therapy)		Health seeking for an urgent health problem		No date-2018		27 (29 papers)			United States (n=9) WHO Europe (n=12) United Kingdom (n=9) Norway (n=1) Netherlands (n=2) N/A (n=1) Not reported (n=5)		Quantitative (n=21) Uncontrolled observational (n=12) Simulation (n=4) RCT (n=2) Experimental audit (n=1) Physician vs symptom checker (n=1) Other (n=1) Qualitative (n=1) Not reported (n=5)	
	Cho et al [23]	Patients aged 18 years and older who are diagnosed with cancer. Includes family members		Electronic symptom self-reporting system or tool		Reporting outside of the clinic or hospital setting		2010-2020		33			United States (n=15) WHO Europe *(n=15)* United Kingdom (n=1) The Netherlands (n=1) Norway (n=1) Denmark (n=1) France (n=3) European countries (n=1) Sweden (n=3) Germany (n=1) Finland (n=2) Other developed (n=2) South Korea (n=2)		Quantitative (n=25) Quasi-experimental (n=17) Experimental (n=7) Case control (n=1) Qualitative (n=1) Mixed methods (n=7)	
	Haridy et al [24]	Patients with chronic viral hepatitis		Telemedicine, electronic medical record s, mobile apps (mHealth^g^), web-based or email intervention, social media, or novel devices		Settings in which screening, diagnosis, or treatment is provided		No date-2020		80			Reported as follows: North America (n=56) Europe (n=10) Australasia (n=7) Asia (n=7)		Quantitative studies (n=51) Observational (n=10) Quasi-experimental pre-post (n=21) RCT (n=3) Cluster randomized (n=2) Retrospective cohort (n=11) Prospective cohort (n=2) Group randomized (n=2)	
	Heaslip et al [25]	Homeless population within underlying health conditions		Mobile phone technology		Homeless accessing any health or warfare services		2015-2017		17			United States (n=13) WHO Europe (n=2) Italy (n=1) United Kingdom (n=1) Other developed (n=1) Canada (n=1)		Quantitative (n=5) Qualitative (n=10) Mixed methods (n=2)	
	Hirvonen et al [26]	Older adults defined as 50-70 years old (majority of study participants had to fall into this age range)		eHealth (eg, web-based personal health records, telehealth services, and mHealth)		Not stated		2010-not stated		32			United States (n=15) WHO Europe (n=10) Netherlands (n=3) United Kingdom (n=3) France (n=1) Spain (n=2) Germany (n=1) Other developed (n=6) Taiwan (n=2) Australia (n=2) New Zealand (n=1) Canada (n=1) LMIC (n=1) Malaysia (n=1)		Quantitative (n=10) Questionnaire (n=8) Nonrandomized (n=1) RCT (n=1) Qualitative (n=14) mixed methods (n=8)	
	Kapadia et al [27]	Includes at least 1 ethnic minority group as health service users		Digital health app and web-based digital information		Web-based NHS^h^ services from primary, secondary, and tertiary care		2011-2021		12			WHO Europe (n=12) United Kingdom (n=4) England (n=7) Scotland (n=10)		Quantitative (n=10) Cross-sectional (n=9) RCT (n=1) Qualitative (n=1) Mixed methods (n=1)	
	Kruse and Heinemann [28]	Patients		Telemedicine in all aspects of care		Put in place during the COVID-19 Pandemic		2020-2021		46			United States (n=16) WHO Europe (n=12) Belgium and Iceland (n=2) Spain (n=2) Sweden (n=1) Czech Republic (n=1) Spain and Netherland (n=1) Finland (n=1) Spain and Netherlands and Taiwan (n=1) United Kingdom (n=1) France (n=1) Netherlands (n=1) Other developed (n=12) Australia (n=5) Canada (n=2) Korea (n=1) China (n=2) Japan (n=1) Taiwan (n=1) LMIC (n=6) Brazil (n=2) Israel (n=1) India, Uganda, and Zimbabwe (n=1) Iran (n=1) Peru (n=1)		Quantitative (n=32) RCT (n=18) Cross-sectional (n=5) Prospective (n=3) Clinical trial (n=2) Posttrial (n=2) Open label intervention (n=1) Cohort (n=1) Nonexperimental (n=1) Qualitative (n=9) Mixed methods (n=3)	
	Litchfield et al [4]	Any individual who uses digital technology in relation to their health and well-being		Health care in the developed world in the early stages of the COVID-19 pandemic		Not stated		2020-2021		9			United States (n=6) WHO Europe (n=3) United Kingdom (n=1) Italy (n=2)		Quantitative (n=8) Cohort (n=6) Cross-sectional (n=2) Mixed methods (n=1)	
	Meirte et al [29]	—^i^		Electronic Patient-reported Outcome Measures questionnaires in a digital form (ie, mobile phone app, tablet, or computer)		Clinical setting		Not date-2017		32			United States (n=11) WHO Europe (n=17) United Kingdom (n=3) Norway (n=1) Austria (n=1) Netherlands (n=5) Germany (n=1) Spain (n=1) France (n=1) Italy (n=2) Switzerland (n=1) Denmark (n=1) Other developed (n=3) China (n=1) Canada (n=2) Unknown (n=1)		Quantitative (n=32) Observational studies (n=14) Experimental studies (n=18)	
	Pang et al [30]	Older adults (>65 y) with cancer. (average sample in the article had to be aged 65 years and older)		Any technology use to aid the delivery of health care		Health care setting		Not date-2020		19			United States (n=7) WHO Europe (n=11) Demark (n=1) Germany (n=2) Netherlands (n=1) United Kingdom (n-7) Other developed (n=1) Canada (n=1)		Quantitative (n=15) Cross-sectional (n=10) Nonrandomized (n=2) Pre- and posttest (n=1) RCT (n=2) Qualitative (n=4)	
	Parker et al [31]	Contains different socioeconomic or disadvantaged groups		Remote GP^j^ consultations		Primary care—GP consultation		No date-2020		13			United States (n=4) WHO Europe (n=8) United Kingdom (n=2) Denmark (n=2) Italy (n=1) Sweden (n=1) Spain (n=1) Netherlands (n=1) Other developed (n=1) Canada (n=1)		Quantitative Retrospective longitudinal studies (n=8) Cross-sectional surveys (n=3) Interrupted time series (n=1) Mixed methods (n=1)	
	Reiners et al [32]	Presence of a chronic disease		eHealth technology for chronic disease		Not stated		2008-2018		22			United States (n=7) WHO Europe (n=8) England (n=2) Germany (n=2) Spain (n=1) Poland (n=1) Sweden (n=1) Netherlands (n=1) Other developed (n=4) Canada (n=1) Australia (n=2) South Korea (n=1) LMIC (n=3) Bolivia (n=1) Malaysia (n=1) India (n=1)		Quantitative (n=20) Nonrandomized (n=11) Descriptive (n=7) RCT (n=2) Qualitative (n=1) Mixed method (n=1)	
	Truong et al [33]	Racial and ethnic minorities of any age, including their care and health care providers		Health care settings		Telehealth consultation for clinical assessment, diagnosis, and management		2005-2020		28			United States (n=23) WHO Europe (n=3) Denmark (n=1) Denmark and Sweden (n=1) United Kingdom (n=1) Other developed (n=1) Australia (n=1) LMIC (n=1) Korea, Vietnam, Cambodia, and Uzbekistan (n=1)		Quantitative (n=19) RCT (n=11) Cohort (n=1) Quasi-experimental (n=2) Cross-sectional (n=4) Case series (n=1) Qualitative (n=3) Mixed methods (n=6)	
	Verma et al [34]	Older adults (≥65 years) living with cancer or a cancer survivor and their care givers		Digital health (eg, technologies with internet, such as smartphones or wearables)		Not stated		2000-2021		11			United States (n=2) WHO Europe (n=3) Denmark (n=2) Germany (n=1) Other developed (n=5) Canada (n=3) Australia (n=2) LMIC (n=1) Iran (n=1)		Quantitative (n=10) Cross-sectional (n=10) Qualitative (n=1)	
	Woodcock [35]	Not stated		Automatic patient self-scheduling		Booking outpatients appointments		No limits		30			United States (n=10) WHO Europe (n=5) England (n=4) 7 countries within WHO Europe (n=1) Other developed (n=10) Taiwan (n=3) China (n=3) Australia (n=3) Canada (n=1) LMIC (n=4) Iran (n=3) Philippines (n=1) Other (review) (n=1)		Quantitative (n=26) Cross-sectional (n=20) Case study (n=3) Case control (n=2) Descriptive (n=1) Mixed methods (n=2) Other (n=2) Systematic review (n=1) Commentary (n=1)	
	Yao et al [36]	General population		Any digital health intervention		Not stated		1990-2020		41			United States (n=19) WHO Europe (n=15) United Kingdom (n=8) Norway (n=3) Italy (n=2) Netherlands (n=1) Switzerland (n=1) Other developed (n=4) Canada (n=2) Australia (n=1) Korea (n=1) LMIC (n=3) Bangladesh (n=1) Indonesia (n=1) Israel (n=1)		Literature reviews (n=6) Quantitative (n=17) Qualitative (n=15) Mixed methods (n=3)	
**Reviews of reviews**																
	Antonio et al [37]	Patients regardless of demographic and disease characteristic Health providers, consumers, educators, policy makers, researchers, and the public		Patient portal Patient web portal Tethered personal health record		Clinical setting in any country except LMIC		1990-2019		14 reviews			Not stated		N/A	
	Barnett et al [38]	Diagnosed mental health condition or receiving mental health care. Include staff and family members of people receiving mental health care		Any spoken or written communication (internet or telephone) between a mental health professional and the patient, family member, service uses, carer, or other health professional		Not stated		2010-2020		19 reviews			Not stated		N/A	

^a^Italics represent main categories (eg, a specific region or a type of research methodology)

^b^WHO: World Health Organization.

^c^N/A: not applicable.

^d^LMIC: low- and middle-income country.

^e^RCT: randomized controlled trial.

^f^The total number of study designs reported by Carini et al [21] is 48 despite only including 47 studies; this is due to 1 study being counted as both an observational study and a descriptive study.

^g^mHealth: mobile health.

^h^NHS: National Health Service (NHS is the umbrella term for the publicly funded health care systems of the United Kingdom).

^i^Not available.

^j^GP: general practitioner.

### Place of Residence

Of the 22 reviews, 3 (14%) concluded that digital technology improved access to health care for rural residents [19,20,24], but these reviews did not consider those who may live in rural areas who do not have access to the digital technology required to use a digital health service. Overall, 14% (3/22) of reviews concluded that the use of digital health care was higher in urban areas [18,21,31]. However, 5% (1/22) of reviews that focused on digital health care among patients with chronic disease reported no difference in use by place of residence [32]. A key reason for lower use in the older adult rural population was lower eHealth literacy levels [34]. No evidence of the difference in engagement with digital technologies by place of residence was identified in the included reviews. There is a lack of research exploring differences in place of residence that considers underlying differences in digital infrastructure, health care provisions or models, and population demographics to clearly ascertain the impact of place of residence on digital health.

### Race, Ethnicity, Culture, Language, and Religion

Access to digital health services among patients from ethnic minority backgrounds with depression was found to be reduced in 5% (1/22) of reviews [38]. Evidence of an effect was observed with the use of digital health technology by ethnicity [4,18,21,22,32,37] and immigration status [31], with 5 reviews [4,18,21,22,37] showing a decreased use by ethnic minorities compared with individuals from a White ethnic background. Although 9% (2/22) of reviews reported no direction [27] or a mixed direction of effect [32], many of the included studies within these reviews showed a greater use among individuals from White ethnic backgrounds.

Mixed evidence was reported for the association between ethnicity and engagement with digital health technology, with 14% (3/22) of reviews showing higher engagement [18,23,32], 5% (1/22) of reviews showing lower engagement by patients from White ethnic backgrounds than ethnic minority backgrounds [33], and 9% (2/22) of reviews finding no conclusive evidence of a difference between ethnic groups [4,27].

Only 5% (1/22) of reviews reported that language may negatively influence access to digital health technology, as patient portals were not offered in the patients’ preferred language [18]. In addition, Litchfield et al [4] and Carini et al [21] noted 1 primary study each reporting lower use of digital health technology among non–English-speaking patients. There is a paucity of research exploring the influence of language barriers on engagement with digital health services. In addition, no evidence was found in the included reviews for culture and religion.

### Occupation

No evidence was identified within the included reviews reporting the impact of occupation on access to digital health care and inconclusive evidence was reported for engagement [23]. Of the 22 reviews, 1 (5%) review concluded that being employed made no significant difference to eHealth use among patients with chronic diseases [32], while Chambers et al [22] found evidence that e-consultation users were more likely to be in employment than nonusers sociodemographic factors, such as gender and sex and age as well as health status, could explain these mixed results [32].

### Gender and Sex

Among the included reviews, there was no evidence of the impact of gender and sex on access to digital health. Of the 22 reviews, 8 (36%) reviews reported on the impact of gender and sex on the use of digital technology, with 4 (18%) reviews indicating consistent evidence of a greater use of digital health technology among women [22,31,35,37]. Of the remaining 18% (4/22) of reviews, 2 observed no difference in use by gender and sex [4,21] and 2 reported mixed evidence [32,39]. There was mixed evidence (2/22, 9%) reported for the association between gender and sex and engagement with digital health, and it was not consistent across all interventions. Men were more likely to accept telemonitoring and electronic self-reported systems than women, but higher satisfaction and engagement were reported in women using eHealth [23,32].

### Education

The association between education and access to digital health was not reported in any of the reviews. However, 18% (4/22) of reviews reported a possible association between the use of digital health care and education showing that individuals with higher levels of education were more likely to use digital health services than individuals with a lower education level [18,21,29,35]. Only 5% (1/22) of reviews reported inconclusive evidence [32]. A higher level of education was also reported to lead to greater engagement with digital health services (2/22, 9%), but this may vary according to the type of technology. For instance, Chambers et al [22] noted that, in 1 primary study [40], individuals with a low to medium level of education were more motivated toward indirect consultation (eg, involving communication with a health professional via email) to reduce uncertainty.

### Socioeconomic Status

Lower income was reported to reduce access to digital health care in 1 included review [32]. However, the association between the use of digital health technology and socioeconomic status was reported in 23% (5/22) of reviews, 4 of which [4,18,35,37] reported higher adoption in higher income than lower income groups and 1 review [31] reported mixed results. Mixed results were also observed with engagement in digital technologies in the included reviews (2/22, 9%) [4,32].

### Social Capital

No evidence on social capital was obtained from the included reviews. Two primary studies [41,42] were identified from an additional search of the literature, with 1 showing better access to televisions among patients with dementia with the presence of a caregiver during the Italian COVID-19 pandemic lockdown in Milan [41]. However, Paccoud et al [42] found no association between social capital (whether an individual knows someone who uses digital technology) and access to or engagement with personal health records but found an association with the use of digital health care.

### PLUS—Other Characteristics

#### Age

Among the included reviews, no evidence was identified for the association between access to digital health and age. However, the association between age and use of digital health was assessed in 12 reviews [4,22,23,26,29,31,34,35,37]. Lower use of digital health technologies in the older population (>50 years) compared with the younger population was identified in 8 reviews [18,22,23,26,29,32,35,37], and 3 reviews found mixed evidence [4,21,31], with lower health literacy being a commonly reported barrier among older people [29,34]. Parker et al [31] observed that the use of different types of digital health technology differed between age groups by Parker et al [31]. For instance, older adults were more likely to use telephone consultations, whereas internet-based consultations were more likely to be used by younger individuals.

Engagement with digital health technology by age was documented in 23% (5/22) of reviews [23,26,30,32,35], with 14% (3/22) of reviews highlighting lower preference or interest among older adults [30,32,35] due to concerns over losing contact with health care professionals [32] and privacy and security concerns [26,30]. However, 5% (1/22) of reviews noted that older adults had greater sustained interest compared with younger adults once they had adopted the technology [26], and another review found no association [23].

#### Disability or Complex Health Needs

The association between disability and access to digital health was reported in 14% (3/22) of reviews [18,29,43], highlighting barriers for individuals with physical, visual, neurocognitive, and intellectual disabilities [18]. The use of digital health technology was observed in 14% (3/22) of reviews [21,36,37], reporting an increased use of patient portals among individuals with high illness burden, depression, moderate-severe asthma, and well-controlled diabetes but lower use among individuals with schizophrenia and schizoaffective disorders. However, we found that use varies depending on health status, the need to be addressed (eg, clinician contact and health information), and the number of comorbidities so that no overall effect could be established [21,37]. No evidence of engagement with digital health technologies was identified in the included reviews.

#### Homelessness or Substance Misuse

Information on access to digital health care by the homeless or substance misuse individuals was not observed in any of the reviews, but details on use (1/22, 5%) [25] and engagement (1/22, 5%) [31] were identified. Heaslip et al [25] found that homeless people are twice as likely to seek health advice on the web if they are using class A drugs, while young homeless people who indicated they had a mental illness were 5 times more likely to seek help in the web. Parker et al [31] found that telephone appointments improved engagement with patients with opioid addiction in primary care compared with face-to-face appointments.

### Result Summary and Evidence Gaps

**Figure 3:**
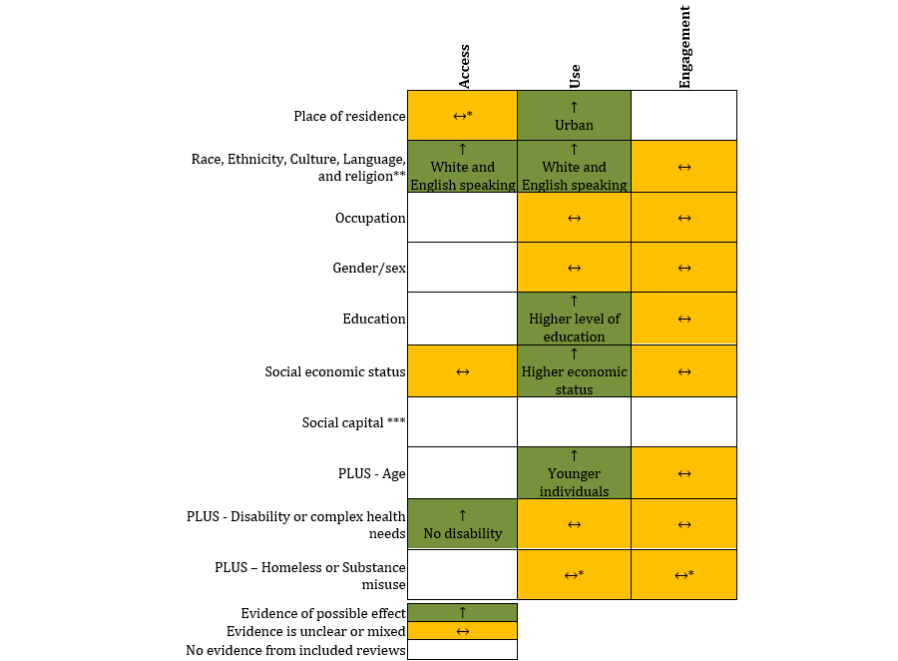
Direction of effect from the evidence obtained from the included review by PROGRESS PLUS. *Some evidence indicated that provision of digital health care increased an individual’s ability to access health care. **For the purpose of this review “Religion” was included in the “race, ethnicity, culture, and language” element of PROGRESS PLUS. ***No evidence was identified within the included reviews for social capital; however, evidence of a mixed effect was obtained from 2 primary studies.

Evidence of a possible effect were evident for access with race, ethnicity, culture, language, and religion and disability or complex needs and for use with place of residence, race, ethnicity, culture, language and religion, education, socioeconomic status, and age (Figure 3). The association between the remaining categories within PROGRESS PLUS with access, use, and engagement had unclear or mixed evidence of a possible effect.

## Discussion

### Principal Findings

This comprehensive scoping review has highlighted inequities among rural communities, ethnic minorities, lower-educated individuals, those with lower socioeconomic status, older adults, and individuals with disabilities or complex needs, with digital technologies across the 3 dimensions of digital health (access, use, and engagement). Owing to the digitalization of health services during the COVID-19 pandemic and their continued use, there remain key policy implications at both an organizational and governmental level to provide equal access to health care via digital platforms for all individuals within society. Given that digital health care has the potential to lower costs, enable prioritization of care, increase adherence to medicine and treatment, and increase self-care [44], there are widespread benefits if barriers to access, use, and engagement can be equitably addressed through policy and practice. In the subsequent sections, we present key inequities found across the 3 dimensions of digital health (access, use, and engagement) and elaborate on key areas for future development in this area. Considering the limitations of this scoping review, our findings and the identified key areas for future development can be used to inform the development and integration of digital health technologies into everyday health care, in an equitable way.

### Strengths and Limitations

We conducted a comprehensive scoping review of equity in digital health technology, collating evidence across 3 key components of digital health (access, use, and engagement) and 10 domains of equity, as defined by the PROGRESS PLUS framework. However, there are some limitations that need to be considered.

Despite there being evidence in the literature for a digital divide in singular domains, such as age, occupation, and sex and gender [5,9,45,46], these divides were mainly reported without considering the interconnectedness of different social classifications (eg, women from ethnic minorities live at the intersection of multiple social, economic, and cultural disadvantages that contribute to being digitally excluded). Examining the intersectionality between access and equity is crucial to protecting against widening inequalities in digital health systems. Furthermore, there was a high level of heterogeneity in the included study populations, which could explain why there was no clear evidence identified within the included reviews for age, occupation, gender and sex, and education. There was also considerable heterogeneity in how types of digital technologies were described (eg, inconsistent and evolving definitions of eHealth modalities, such as patient portals).

No evidence could be obtained from other reviews on social capital and religion. These elements of PROGRESS PLUS are more likely to be explored in qualitative research [27]. More research examining ethno-religious groups would increase our understanding of how diverse factors such as age, religion, and gender interact and contribute to a digital divide [27,41,42]. This review focuses on providing evidence for the WHO European region, but many of the studies documenting race, ethnicity, and language come from US studies, which potentially limits it generalizability to the European context. Furthermore, the evidence within Europe is dominated by Western European countries and the situational context may be different in countries that are classified as LMIC.

Finally, only quantitative studies were investigated, and future work is required to capture evidence for qualitative studies to provide a greater understanding of the current facilitators and barriers to digital health care. Nonetheless, a validation check of the results of this review against those of qualitative reviews revealed similar findings. Methodological weakness observed by those reviews that undertook a critical appraisal (12/22, 55%) should be addressed in any future work, these include low participation rate, small sample size, unblinded participants, lack of control for confounding, and biased samples that only included individuals with access to digital technologies. Another important bias that needs to be considered is the underrepresentation of non-English speakers and ethnic minorities in current literature [47]. Efforts to include these population groups are needed to develop culturally informed digital health technologies to address their needs.

### Comparison With Prior Work

#### Access

The ability to access the resources required for digital health differs by ethnic group and disability or complex health needs status, a finding that is consistent with the literature [48-50]. However, there was unclear evidence for socioeconomic status and place of residence, despite rural areas having a higher rate of digital exclusion compared with urban areas [2] owing to access and connectivity issues [19,20]. It should also be noted that access to digital technology is not homogenous across the WHO European region, with greater access in North-Western Europe compared with Eastern or Southern Europe [2]. Mapping digital infrastructure and inequities in access, including pay barriers, could help identify gaps and support policy and intervention decisions to support digital health.

#### Use

Individuals from different places of residence, ethnicity, education level, socioeconomic status, and age were found to vary in their ability to access and use digital health technology. Many of the studies within the included reviews reported a greater use of digital health technologies among patients of White ethnic background, with ethnic minorities facing greater barriers to health care at an individual, provider, and system level [51,52]. These barriers to the use of digital health care include a lack of alternative language formats and lower digital literacy [33]. The lack of digital skills is also present as a barrier for older populations [29,34]. A failure to consider and adjust for underlying factors, such as sociodemographic characteristics [22], structural inequalities, and important confounding factors (eg, literacy rate and language skills), may explain the lack of clear evidence in some of the PROGRESS PLUS categories (eg, occupation, gender, sex, and disability).

#### Engagement

No clear direction of effect was observed within any of the PROGRESS PLUS categories for engagement in digital health technologies. Having high access and ability to use digital health technologies does not necessarily translate into being more motivated or interested in engaging with digital technologies [53]. Barriers identified include security concerns [37], mistrust [54], preference for face-to-face health care interventions [27,55], and concerns that face-to-face health care interventions will cease [11]. Understanding and exploring ways to increase engagement among vulnerable members of society is key to reducing digital health inequity, especially when there are increasing demands and pressures on health services. It is possible that qualitative data would provide a richer understanding of the barriers for user engagement and help the development of digital health care resources that address users’ needs.

#### Suggestions for Future Work, Solutions, and User-Centered Approaches

Within this scoping review, it was difficult to draw conclusive evidence for all elements of PROGRESS PLUS. Reasons include the heterogeneity of the evidence, methodological weaknesses, and examination of singular domains of equity, resulting in a lack of investigation into intersectionality in the current literature. Further high-quality research is required to address key evidence gaps identified in this scoping review. This will increase our understanding of key barriers in access, use, and engagement across different regions within the WHO European region and across underrepresented population groups that need to be targeted at a policy level. We also recommend following a systematic approach to reporting the population studied to help enable more rapid learning in digital health innovation and inequalities. This systematic approach should consider how digital health should be evaluated at the design stage and ensure effective data capture during implementation [56]. Incorporating intersectional analysis into research in this area will increase our understanding of the key drivers contributing to equity in digital health and would account for the complex systems at play [47,57-59]. We recommend developing a common reporting framework to monitor and collate high-quality evidence and developing a structured approach to the evaluation of digital health initiatives against equity domains. This, in turn, would support decision-making in this area and facilitate knowledge exchange at a national and regional level.

Several reviews highlighted the importance of digital health technologies addressing the user needs in both content and design [21,26,29,31,32,37]. Given this, digital solutions should be designed with inclusive and participatory user-centered approaches (co-design and coproduction) to ensure that technology use is appropriate and adds value to the end user [60]. This is particularly important for those considered as at-risk populations, such as those with disabilities and complex needs, and those experiencing language barriers.

Digital skills are a key limitation in using digital health care [29,34]. Therefore, equipment, training, and educational resources for professionals and end users are required to increase adoption [61], perhaps in the form of community and patient hubs [44]. For example, disadvantaged groups could be supported through training and provision of dedicated internet connections and digital devices within the community [28]. Consideration must also be given to the medium in which these resources are presented [27,28] and ensure information is simplified and accessible [34]. There are opportunities to capitalize on “quick wins,” such as language-related solutions that could enable access to patients from ethnic minorities without the need for large infrastructure adjustments. Furthermore, creation of protocols and regulations around the privacy and security of digital health technology in accessible formats could address privacy and safety concerns [29,62].

Finally, any research and evaluation into digital health inequities need to be relevant to policy and practice and link in with WHO Europe’s digital health action plan [63]. Collective approaches among local, national, and international organizations, including the creation of a common definition of digital health, such as updating the WHO digital health classification tool [64], could help in monitoring and reporting digital inequities. This would also help develop a good practice approach when generating policy-relevant evidence. For example, the NICE (National Institute for Health and Care Excellence) has a standard framework for digital health technologies that cover design, value, performance, and deployment [65], with equity being a standard requirement. However, the fact that not all populations are homogeneous (eg, the urban-rural divide and diverse populations) needs to be considered and a mix of regional and national policy to ensure equity in digital health may be required.

On the basis of our findings, the key areas suggested for future development are as follows:

1. Understand key barriers to access, use, and engagement with digital health technologies across the WHO European region, considering the situational context between regions to reduce bias toward high-income Western European countries and underrepresented population groups through high-quality research.
2. Develop and adopt a common framework approach to monitor and report differences, with a shared digital health definition across all equity domains that collate evidence to inform action.
3. Identify and address potential barriers through mapping inequities in digital infrastructure, understanding the impact of intersectionality and approaches to improve knowledge, skills, and confidence.
4. Ensure interventions are co-designed, inclusive, and participatory, with appropriate evaluation and reporting.
5. Share and collate examples of best practices taken by health care systems to address digital health inequities.

### Conclusions

Within the context of the WHO European region, this scoping review has highlighted present inequities and evidence gaps across multiple domains with digital health care technologies. Rural communities, ethnic minorities, lower-educated individuals, those with lower socioeconomic status, older adults, and individuals with disabilities or complex needs face digital health inequality, and active approaches should be taken to reduce this gap. These active approaches could include the development and adoption of a common framework to support policies and procedures within the WHO European region for current and new co-designed digital solutions, in addition to mapping inequities, investigating intersectionality, and taking into consideration regional differences within the development stage of any initiative. These recommendations could be achieved through further robust research and evaluation where there is currently mixed evidence and the sharing of good practices to create sustainable solutions to reduce digital health inequalities.

## Appendix

Multimedia Appendix 1 PsycINFO search strategy.

## Abbreviations

LMIC: low- and middle-income country
NICE: National Institute for Health and Care Excellence
PRISMA-ScR: Preferred Reporting Items for Systematic Reviews and Meta-Analyses extension for Scoping Reviews
WHO: World Health Organization
