# Supplementary material for: Mapping Inequities in Digital Health Technology Within the World Health Organization’s European Region Using PROGRESS PLUS: Scoping Review
Source: J Med Internet Res. 2023 Apr 28;25:e44181. doi: 10.2196/44181 (PMC10182469; doi:10.2196/44181)
Supplement: Multimedia Appendix 1 [file jmir_v25i1e44181_app1.docx]

| **Set#** | **Searched for** | **Results** |
| --- | --- | --- |
| S1 | (SU.EXACT.EXPLODE("Electronic Health Services")) | 14931 |
| S2 | (SU.EXACT("Information and Communication Technology" OR "Computer Mediated Communication")) | 16950 |
| S3 | (ti,ab("health IT" OR "health information technology" OR "health information technologies" OR ehealth OR eHealth OR "electronic health" OR mhealth OR m-health OR "mobile health")) | 8400 |
| S4 | (ti,ab("digital health" OR telehealth OR telecare OR telemedicine OR teleHealth OR tele-care OR tele-medicine)) | 4740 |
| S5 | (ti,ab((medical or clinical or health or healthcare or nurs*) N/3 informatics)) | 499 |
| S6 | (ti,ab(health N/1 (app OR apps OR application*))) | 1532 |
| S7 | S6 OR S5 OR S4 OR S3 OR S2 OR S1 | 39481 |
| S8 | (SU.EXACT ("Text Messaging" OR "Computer Mediated Communication" OR "Mobile Applications") OR SU.EXACT.EXPLODE("Mobile Devices")) | 10158 |
| S9 | (ti,ab(cell* N/1 (phone* or telephone* or technolog* or device*)) OR ti,ab(mobile* N/1 (phone* or telephone* or technolog* or device*))) | 10604 |
| S10 | (ti,ab(digital N/1 (technolog* or device*))) | 4130 |
| S11 | (ti,ab(smartphone* or smart-phone* or ipad* or i-pad*)) | 7522 |
| S12 | (ti,ab(tablet N/1 (device* or computer*))) | 810 |
| S13 | (ti,ab(electronic mail* OR email* OR e-mail)) | 10893 |
| S14 | (ti,ab((text* OR voice OR electronic OR instant) N/1 messag*) OR ti,ab((text* OR voice OR electronic OR instant) N/1 remind*)) | 3964 |
| S15 | (ti,ab(texting or texted)) | 1092 |
| S16 | (ti,ab(sms N/1 (service* or messag*)) OR ti,ab(mobile N/1 (app OR apps OR application*))) | 3537 |
| S17 | S16 OR S15 OR S14 OR S13 OR S12 OR S11 OR S10 OR S9 OR S8 | 37385 |
| S18 | (ti,ab(Health OR healthcare OR “health care”)) | 666635 |
| S19 | S18 AND S17 | 8787 |
| S20 | S19 OR S7 | 45011 |
| S21 | (SU.EXACT("Healthcare Disparities" OR “Health Equity”)) | 3700 |
| S22 | (MJSUB.EXACT("Rural Health") AND ti,ab(equity or inequit* or equality or inequalit* or disparit*)) | 66 |
| S23 | (ti,ab((health) N/2 (equity or inequit* or equality or inequalit* or disparit*)) OR ti,ab((healthcare) N/2 (equity or inequit* or equality or inequalit* or disparit*)) OR ti,ab(“health care” N/2 (equity or inequit* or equality or inequalit* or disparit*))) | 15483 |
| S24 | (ti,ab("medically underserved area" OR "physician shortage area" OR "underserved patients")) | 135 |
| S25 | S24 OR S23 OR S22 OR S21 | 17784 |
| S26 | (SU.EXACT.EXPLODE("Socioeconomic Factors") OR SU.EXACT("Gender Equality")) | 123141 |
| S27 | (SU.EXACT("Social Equity" OR "Equity" OR "Racial Disparities" OR “social deprivation”)) | 5177 |
| S28 | (SU.EXACT("Immigration" OR "Racial and Ethnic Groups" OR "Refugees" OR "Older Adulthood") AND ti,ab(equity or inequit* or equality or inequalit* or disparit*)) | 3525 |
| S29 | (ti,ab(social N/1 determinant*)) | 4555 |
| S30 | (ti,ab(socioeconomic N/1 (determinant* OR factor*)) OR ti,ab(socio-economic N/1 (determinant* OR factor*))) | 4157 |
| S31 | (ti,ab(rural N/1 communit*)) | 6259 |
| S32 | ti,ab((vulnerable OR underserved OR “low income” OR rural OR sensitive OR disadvantaged) N/1 population*) | 11774 |
| S33 | S32 OR S31 OR S30 OR S29 OR S28 OR S27 OR S26 | 151175 |
| S34 | S33 AND S18 | 51362 |
| S35 | (SU.EXACT("Meta Analysis" OR "systematic review")) | 60762 |
| S36 | (ti,ab(review N/2 (systematic or rapid or scoping or mapping))) | 47129 |
| S37 | ti,ab(meta-analysis OR metaanalysis OR meta-analyses OR metaanalyses) | 42097 |
| S38 | S37 OR S36 OR S35 | 84088 |
| S39 | S34 OR S25 | 62712 |
| S40 | (S39 AND S38 AND S20) and (pd(2016-2022)) | 52° |
